# DNA double-strand breaks induce H2Ax phosphorylation domains in a contact-dependent manner

**DOI:** 10.1038/s41467-020-16926-x

**Published:** 2020-06-22

**Authors:** Patrick L. Collins, Caitlin Purman, Sofia I. Porter, Vincent Nganga, Ankita Saini, Katharina E. Hayer, Greer L. Gurewitz, Barry P. Sleckman, Jeffrey J. Bednarski, Craig H. Bassing, Eugene M. Oltz

**Affiliations:** 1 Department of Microbial Infection and Immunity, The Ohio State University, Columbus, OH 43210 USA; 2 Department of Pathology and Immunology, Washington University School of Medicine, St. Louis, MO 63110 USA; 3 Department of Biomedical and Health Informatics, Children’s Hospital of Philadelphia, Philadelphia, PA 19104 USA; 4 Department of Medicine, Division of Hematology and Oncology, O’Neal Comprehensive Cancer Center, University of Alabama at Birmingham, Birmingham, AL 35294 USA; 5 Department of Pediatrics, Washington University School of Medicine, St. Louis, MO 63110 USA; 6 Department of Pathology and Laboratory Medicine, Children’s Hospital of Philadelphia, Perelman School of Medicine, University of Pennsylvania, Philadelphia, PA 19104 USA

**Keywords:** Epigenetics in immune cells, Chromatin structure, Non-homologous-end joining

## Abstract

Efficient repair of DNA double-strand breaks (DSBs) requires a coordinated DNA Damage Response (DDR), which includes phosphorylation of histone H2Ax, forming γH2Ax. This histone modification spreads beyond the DSB into neighboring chromatin, generating a DDR platform that protects against end disassociation and degradation, minimizing chromosomal rearrangements. However, mechanisms that determine the breadth and intensity of γH2Ax domains remain unclear. Here, we show that chromosomal contacts of a DSB site are the primary determinants for γH2Ax landscapes. DSBs that disrupt a topological border permit extension of γH2Ax domains into both adjacent compartments. In contrast, DSBs near a border produce highly asymmetric DDR platforms, with γH2Ax nearly absent from one broken end. Collectively, our findings lend insights into a basic DNA repair mechanism and how the precise location of a DSB may influence genome integrity.

## Abstract (web summary)

Formation of γH2Ax serves as a checkpoint for double-strand break (DSB) repair pathways. Here the authors reveal via integrated chromatin analysis that γH2Ax domains are established by chromosomal contacts with the DSB site.

## Introduction

All cells continuously face DNA damage resulting from environmental insults or from normal physiological processes, including replication and transcription. Perhaps the most dangerous type of damage to DNA is double-strand breaks (DSBs), since their aberrant repair can produce oncogenic rearrangements^1^. When DNA damage occurs in mammalian cells, DSB sensors activate the serine-threonine kinases ATM, ATR, and DNA-PKc^2^, which initiate the DNA Damage Response (DDR) via phosphorylation of ~900 protein targets^3^. An important chromatin-based substrate for these kinases is the histone variant H2Ax that, when phosphorylated on serine 139, is referred to as γH2AX^4,5^. Formation of γH2Ax serves as a checkpoint for the homologous recombination (HR) and nonhomologous end joining (NHEJ) repair pathways, through mechanisms that indirectly or directly retain effector proteins^6,7^. These effectors include 53BP1, which prevents end degradation and disassociation^8–10^. Indeed, H2Ax deficiency destabilizes chromosomes harboring DSBs^11^, leading to numerous aberrations, including translocations and deletions^12,13^.

Pursuant to a break, the DDR generates γH2Ax domains that are thought to spread over neighboring chromatin for 1–2 Mb, perhaps by propagation along the chromosome^14–16^. However, a classic, processive model cannot fully explain the observed profiles of γH2Ax, which can be asymmetric, and may have gaps and varying levels of the modification throughout a domain^11,15,17^. Likewise, γH2Ax foci are not contiguous when visualized by high-resolution microscopy, which revealed spatially distinct nano-domains clustered around DSB sites^18^. The mechanisms that sculpt γH2Ax domains have important implications, especially given the critical role of these platforms in damage responses, including: (1) tethering broken chromosomes until they are repaired^11,19^ (2) repression of transcription^20,21^, and (3) sequestration of DDR factors around a DSB site^18,22^.

Prior studies have shown that perturbation of DDR mechanisms, including mutations in ATM and MDC1 alter γH2Ax densities, but do not affect the extent of its spread^17^. We now show that γH2Ax domains are established via chromosomal contacts with the DSB site. Indeed, the break site interactome precisely defines the densities and spread for this damage-induce histone modification. γH2Ax domains are largely, but not exclusively, confined within self-interacting chromatin regions, called topologically associated domains (TADs)^23^, which functionally compartmentalize the genome. Disruption of a TAD border by a targeted DSB extends γH2Ax domains into both adjacent TADs. In contrast, DSBs adjacent to TAD borders generate asymmetric γH2Ax domains, which may influence repair efficiencies and could explain the enrichment of structural variants near topological boundaries.

## Results

### Physiologic DNA breaks induce locus-restricted γH2Ax domains

**Fig. 1:**
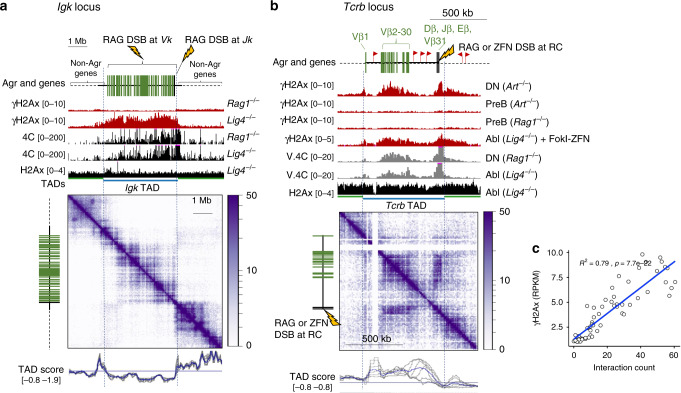
H2Ax phosphorylation is confined to antigen receptor loci following RAG-mediated DSBs. Genome browser snapshots of the **a** *Igk* or **b** *Tcrb* antigen receptor regions. Each panel includes diagrams indicating antigen receptor loci, genes, and DSB location (lightning bolt) on top. **a** *Igk* locus snapshot of data derived from G1-arrested v-abl pre-B cells following Imatinib treatment (72 h). UCSC genome browser tracks show RPKM-normalized histograms for γH2Ax ChIP-seq (mean of three independent replicates), *Jk* interactome 4C-Seq (representative of two independent replicates), and H2Ax ChIP-seq (representative of two independent replicates) for indicated v-abl cell genotypes. The bottom Juicebox snapshot shows Hi-C data derived from G1-arrested *Lig4*^−/−^ cells (*n* = 2, merged independent samples). Scale indicates interaction counts. The IGV tracks under the Hi-C data show topologically associated domains (TADs), which are assigned based upon TAD insulation scores, derived from 40 kb bins (blue lines) or 20–100 kb bins (gray lines). **b** *Tcrb* locus snapshot with data derived from both primary and G1-arrested v-abl cells. The UCSC genome tracks show RPKM-normalized histograms for γH2Ax CR-seq (*n* = 2, representative of independent replicates), *Tcrb*-RC viewpoint virtual V.4C-seq (*n* = 2, merged independent replicates), and H2Ax ChIP-seq (representative of two independent replicates) for indicated Abl cell genotypes. To generate V.4C tracks, 10 kb-binned Hi-C data were extracted for the RC viewpoint and plotted. Bottom: Hi-C Juicebox plot derived from *Rag*^*−/−*^ DN thymocytes (*n* = 2, merged independent samples). The tracks under the Hi-C data show TADs and their insulation scores as in **(a**). **c** DN thymocyte Pearson’s correlation of γH2Ax RPKM and interaction counts in 5 kb windows across *Tcrb*.

To probe genomic features that limit γH2Ax propagation, we characterized DDR platforms in precursor lymphocytes resulting from physiological DSBs, which are mediated by the RAG endonuclease complex during V(D)J recombination. Initially, we profiled chromatin following RAG-induced DSBs at the *Igk* antigen receptor locus in G1-arrested v-abl transformed pre-B cells^24^. We employed a particular line of v-abl cells in which RAG breaks are persistent due to a crippling mutation in the essential NHEJ gene, *Lig4*^25^. In *Lig4*^−/−^ cells, but not in control *Rag1*^−/−^ cells (Lig4^wt^), which lack *Igk* breaks, γH2Ax covered the entire *Igk* locus, as revealed by chromatin immunoprecipitation (ChIP)-seq analysis (Fig. 1a, *p* < 0.01 Fisher’s Exact Test). The boundaries of the γH2Ax domain, as well as the *Igk* locus, coincided with the encompassing TAD, as computed from global interactomes in *Rag1*^*−/−*^ cells (Fig. 1a and Supplementary Fig. 1A). Moreover, γH2Ax profiles correlated with the magnitude of chromosomal contacts measured by 4C from the viewpoint of the small *Jk* cluster, which always harbor a DSB in the v-abl system (*R* = 0.60, Pearson’s correlation). Overall patterns in the *Jk* interactome did not differ substantially in cells with (*Lig4*^−/−^) or without *Igk*-DSBs (*Rag1*^−/−^), as revealed by 4C analysis (Fig. 1a). Importantly, contours and borders of the RAG-induced γH2Ax domain did not reflect, at a gross level, those of the un-phosphorylated histone substrate, H2Ax (Fig. 1a). However, in addition to the small *Jk* cluster, the RAG complex targets DSBs to synapsed *Vk* gene segments, which are distributed throughout the 2.5 Mb *Vk* cluster. As such, profiles of γH2Ax within *Igk* may simply correspond to a broad distribution of DSBs throughout the *Vk* cluster in this pre-B cell population.

To circumvent this complication, we examined γH2Ax patterns in thymocytes from Artemis-deficient, Bcl2-Tg mice, which harbor persistent, RAG-mediated DSBs at the *DbJb* recombination center (RC) of *Tcrb*^26,27^. In this case, we employed native Cut and Run sequencing (CR-seq), which, importantly, avoids potential artifacts associated with chromatin crosslinking in conventional ChIP-seq^28^. As shown in Fig. 1b, γH2Ax spread throughout most of the *Tcrb* locus, despite confinement of the DSBs to its 3′RC portion. In these primary cells with *Tcrb* damage, γH2Ax values in CR-seq data correlated almost precisely with RC chromosomal contacts in *Rag1*^−/−^ thymocytes, which we defined quantitatively using deep Hi-C data that were flattened to show the RC viewpoint (herein called virtual 4C, V.4C; Fig. 1b, c, *r* = 0.89 Pearson’s correlation). A nearly identical γH2Ax profile was observed in *Lig4*^−/−^ v-abl cells when we used a FokI zinc finger nuclease (FokI-ZFN) to target the *Tcrb*-RC with a DSB^25^, thus indicating that the γH2Ax domains were independent of the initiating nuclease. Indeed, γH2Ax intensities correlated almost precisely with contacts observed for the DSB site (Fig. 1c). Moreover, γH2Ax domains correlated with DSB-site interactomes at two other *Tcr* loci harboring RAG breaks in Artemis-deficient, Bcl2-Tg thymocytes, namely *Tcrg* and *Tcra/d* (Supplementary Fig. 1B, C). We conclude that DSBs within antigen receptor loci, induced by either RAG or designer endonucleases, produced γH2Ax domains whose widths and densities tracked closely with the interactomes of the break sites.

### γH2Ax profiles parallel cell type-specific contacts

**Fig. 2:**
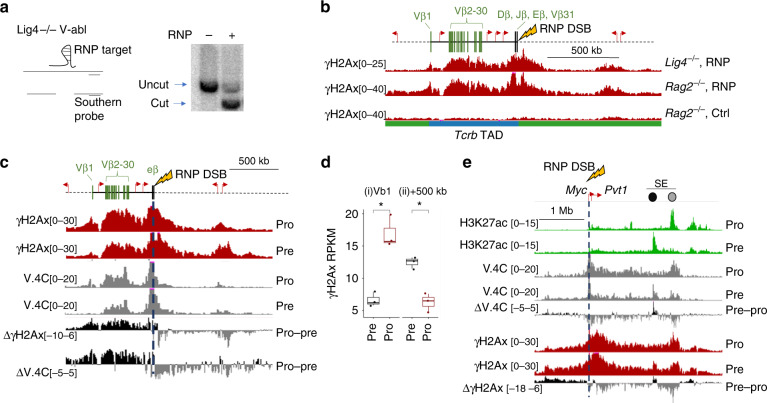
Contact-dependent γH2Ax profiles in a tractable cell model. **a** Southern blotting analysis for DSBs targeted to the Eβ enhancer in *Tcrb*. Bands corresponding to uncut (top band) or cut (bottom band) loci are indicated. Genomic DNA was harvested 4 h after *Lig4*^−/−^ or *Rag2*^−/−^ v-abl cells were nucleofected with an RNP targeting Eβ. See Supplementary Fig. 2A for replicates. **b** UCSC genome browser tracks showing γH2Ax CR-seq performed 2 h after *Lig4*^−/−^ or *Rag2*^−/−^ v-abl cells were nucleofected with RNP-Eβ (*n* = 2, representative of independent replicates). *Tcrb* gene segments, neighboring genes (red arrows) and the DSB location (lightning bolt) are shown at the top. TAD locations are indicated on bottom. **c** UCSC genome browser tracks showing the *Tcrb* loci in pro- (63–12 cell line) or pre-lymphocyte cell lines (p5424). For each panel, the locations of gene segments, regulatory elements, and RNP target (lightning bolt and dashed line) are shown at the top. Tracks represent values for γH2Ax CR-seq (red, RPKM, *n* = 3, representative of independent replicates), V.4C (gray, interaction count, *n* = 2 merged independent replicates). In panels with interactome data, V.4C is extracted from the DSB site (dark blue line). **c**, **e** include subtraction plots (labeled Δ) for γH2Ax and V.4C data, showing differences in (mean of independent replicates) signal from pro- versus pre-lymphocytes. **d** Box and whisker plot showing γH2Ax in 25 kb bins after pre- (p5424: gray) or pro- (63–12: gray) lymphocyte cell lines were nucleofected with an RNP-Eβ. Data points show three biologically independent replicates. Means, quartiles, and outlier limits (1.5 × interquartile range) are indicated by the median line, box and whiskers, respectively. Relative locations are indicated above the graph. *p < 0.05, two-sided Paired Student’s T test. Enrichment and statistics for all bins across the *Tcrb* locus is shown in Supplementary Fig. 2C. **e** UCSC genome browser tracks showing the *Myc* locus, as in (**c**). Green tracks represent H3K27ac CR-seq (green, RPKM, *n* = 1). Cell type-specific H3K27ac+ regions near the Myc Super-Enhancer (SE) are indicated by colored dots, showing positions for pre- (black) or pro- (gray) lymphocyte enhancers.

To define determinants for DSB-induced γH2Ax domains, we designed a flexible experimental platform, targeting the Cas9 endonuclease with guide RNAs in preformed ribonuclear particles (RNPs), which were delivered into cells by nucleofection. We validated the system by targeting Cas9 breaks to the Eβ enhancer in G1-arrested *Lig4*^−/−^ cells. The Eβ-proximal DSB accumulated to near maximum levels (>80%) within 2 h following RNP nucleofection (Fig. 2a and Supplementary Fig. 2A, B). As shown in Fig. 2b, the Cas9 system recapitulated γH2Ax profiles observed for either RAG- or ZFN-induced DSBs in the *Tcrb*-RC (compare to Fig. 1b). Likewise, in cycling, repair-sufficient cells (*Rag2*^−/−^), which undergo continual cycles of cut and repair, targeted Cas9-DSBs at Eβ produced a nearly indistinguishable DDR platform. Hence, γH2Ax profiles arising from DSBs within the *Tcrb-*RC were identical across distinct cellular sources and experimental systems that had comparable *Tcrb* interactomes.

If the interactome of a break site is the primary determinant for γH2Ax profiles, one would predict that cell types with different interaction magnitudes would produce DDR platforms with distinct contour densities. Indeed, largely tracking with distinctions in their Eβ interactomes, a targeted DSB at this enhancer generated significantly higher levels of γH2Ax across distal Vβ gene segments in a pro-lymphocyte cell line (63–12) when compared with a pre-lymphocyte line (p5424) (Fig. 2c, d and Supplementary Fig. 2C)^29,30^. These differences are highlighted in subtraction plots for both γH2Ax and V.4C data (bottom tracks). Conversely, the pre-lymphocyte cell line exhibited elevated interactions between the *Tcrb*-RC and a +500 kb gene cluster, located in an adjacent TAD, which was reflected in significantly higher downstream γH2Ax intensities (Fig. 2d and Supplementary Fig. 2C).

We observed similar results at an independent locus containing the proto-oncogene *cMyc*, which can adopt cell type-specific topologies^31^. As shown in Fig. 2e, the *Myc* promoter region established long-range contacts with an H3K27ac-dense region, the *Myc* super-enhancer (*Myc*-SE), situated ~1 Mb away^32^. Long-range chromatin contacts between the Myc promoter and enhancers that compose the *Myc*-SE change during development or differentiation, as distinct regulatory regions become activated or are decommissioned^33^. In keeping with this, pro- and pre-lymphocyte lines preferentially activated and formed promoter contacts with distinct enhancer elements in the large *Myc*-SE region (Fig. 2e, HiCcompare *p* < 0.05, *M* test). Although the γH2Ax borders were similar in both cell types, DSB induction at the *Myc* promoter (RNP-*Myc*-P) generated relative changes in γH2Ax densities that mirrored differences in cell type-specific promoter contacts. We conclude that γH2Ax intensities in response to a DSB can differ between cell types in a contact-dependent manner.

We also performed complementary studies to examine the impact of a DSB on chromosomal contacts. For this purpose, we generated Hi-C contact maps for G1-arrested *Lig4*^−/−^ cells harboring persistent DSBs at *Myc* or *Tcrb* (Supplementary Fig. 3A). In both cases, DSBs did not grossly alter the contours of interaction profiles, relative to those receiving either no RNP or an RNP that targets a different chromosome. However, DSBs induced a modest, but significant, enhancement of intra-locus contacts (Supplementary Fig. 3B), a finding consistent with those obtained in cycling cells using a restriction endonuclease to introduce DSBs at naturally occurring sites in the genome^34^, as well as 4C data shown in Fig. 1a. Thus, we conclude that DSBs generate γH2Ax domains through chromosomal contacts, which are enhanced following the lesion, but whose regional profiles and distributions do not change significantly.

### Interactomes rather than TADs limit DDR platforms

**Fig. 3:**
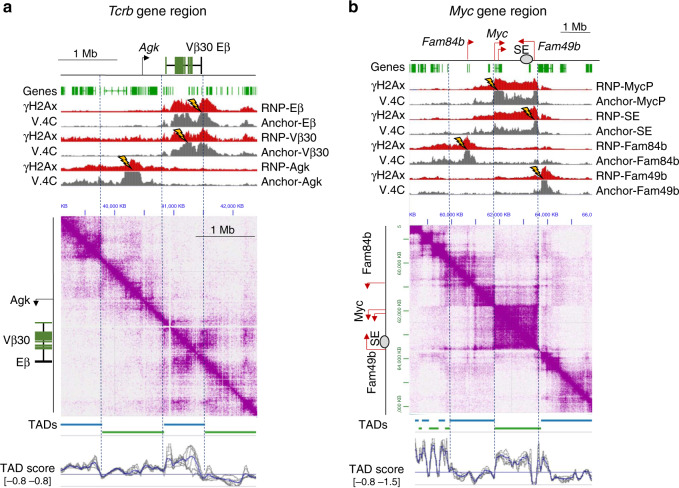
TADs contribute to compartmentalization of γH2Ax signaling domains. JuiceBox browser snapshots showing paired γH2Ax and interactome measurements across *Tcrb* (**a**) or *Myc* (**b**). Locations of genes, gene segments, and regulatory elements are shown at the top of each panel. RNP target locations and V.4C data extraction viewpoints are shown as lightning bolts. Tracks represent values for γH2Ax CR-seq (Red, representative of two independent replicates each, scaled min to max) or V.4C (Gray, *n* = 2 merged independent replicates, 0–25 contacts). Bottom: JuiceBox Hi-C plots after coverage normalization (*n* = 2, merged independent replicates each). G1-arrested *Lig4*^−/−^ v-abl cells were used for *Tcrb* DSBs, while cycling *Rag2*^−/−^ cells were used for targeting DSBs to *Myc*. Dashed lines represent selected TAD locations. The IGV tracks under the Hi-C data also show TADs, as well as their insulation scores, derived from 40 kb bins (blue lines) or 20 to 100 kb bins (gray lines).

If γH2Ax propagates when chromatin interacts with a DSB, we reasoned that nearly any RNP targeted to a single self-interacting region (i.e., a TAD) would generate DDR profiles with similar boundaries. To test this hypothesis, we computationally defined TADs by measuring insulation scores across a series of 10–200 kb bins (Fig. 3a, b, bottom)^35^. Next, we targeted DSBs to two distinct locations within the *Tcrb*-TAD (Vβ30 and Eβ). As shown in Fig. 3a, both lesions produced γH2Ax profiles that paralleled the contours of their interactomes, including large, intra-TAD gaps, and a sharp γH2Ax boundary at the 5′ TAD border. However, both DSBs also generated a γH2Ax region at a gene cluster in the adjacent 3′ TAD, with which the break sites also formed significant contacts. Thus, in *Tcrb*, the DSB interactome, rather than strict TAD borders, served as the primary determinant of γH2Ax boundaries. Likewise, introduction of DSBs at either the 5′ (*Myc*-P) or 3′ (*Myc*-SE) end of the *Myc*-TAD generated robust γH2Ax domains that were contact dependent and largely restricted to the *Myc*-TAD, but also extended into adjacent TADs (Fig. 3b). Strikingly, the two *Myc* DSBs generated highly asymmetric γH2Ax domains relative to the break sites, which were situated on the extreme ends of the *Myc*-TAD (i.e., producing one long and one short γH2Ax domain, see “Discussion”).

Importantly, DSBs at sites in topological domains adjacent to either *Tcrb* (*Agk*) or *Myc* (*Fam84b* and *Fam49b*), produced γH2Ax domains that were largely restricted to their distinct TADs, but also consistently exhibited inter-TAD deposition of γH2Ax that reflected the DSB interactome (Fig. 3a, b). Persistent DSBs at three randomly chosen genomic locations also generated γH2Ax domains whose termini coincided well with those of their parent interactome, and whose (a)symmetries reflected the DSB location relative to adjacent TAD borders (Supplementary Fig. 4). We conclude that interactomes are the primary determinants of DDR platforms, and that TAD borders can impede, but may not completely block, the spread of γH2Ax into neighboring domains.

### Deletion of a CTCF motif reduces γH2Ax spread within a TAD

**Fig. 4:**
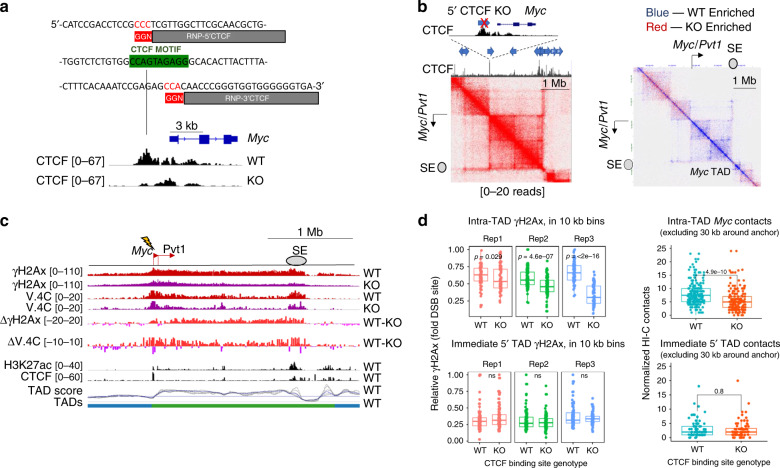
CTCF is necessary for robust γH2Ax propagation in the *Myc*-TAD. **a** CTCF motif deletion within a TAD border upstream of *Myc*. Top: Sequence of the upstream *Myc*-CTCF (green) and RNP target sites (gray and red). RNPs labeled 5′ and 3′ CTCF were used for deletion. Bottom: *Myc* locus in 5′CTCF WT or KO pre-lymphocyte cells (63–12). *Myc* exons and UTRs are shown (top). Tracks represent CTCF CR-seq values (black, RPKM, *n* = 1) and the arrow indicates the CTCF motif. **b** Interactome of 5′CTCF KO cells. Top left insert shows CTCF ChIP-seq data (0–60 RPKM, *n* = 1), CTCF motif orientation (blue arrows), TAD border, and deletion site (red X). Bottom: JuiceBox Hi-C plots show the wild-type *Myc* locus (left) or relative Hi-C interactome changes following CTCF deletion (right) (*n* = 1, each). Blue points are ≥2 interaction counts higher in WT; red points are ≥2 counts higher in the 5′CTCF KO. The location of the *Myc*-TAD is indicated on bottom. **c** UCSC snapshot, as described in Fig. 2, showing the *Myc* locus in 63–12 cells with and without the 5′ CTCF binding motif (representative of three independent results). For subtraction plots of γH2Ax or V.4C data, red represents WT enrichment, while purple represents KO enrichment. **d** *Myc* promoter interactome and γH2Ax derived from 5′CTCF WT or KO cell lines, quantified in 10 kb bins across either the *Myc*-containing TAD or immediate 5′ TAD. Left: γH2Ax 2 h after 5′CTCF WT or KO cells were nucleofected with RNP *Myc*-P. Each γH2Ax dataset was first normalized to RPKM values at the DSB-containing bin. Colors represent biologically independent replicates (*n* = 3). Right: Comparison of *Myc* promoter interactomes, derived from Hi-C data, across the *Myc*-containing TAD or immediate 5′ TAD in cells of indicated genotypes, without RNP nucleofection. Relative interactome measurements have been normalized using coverage normalization. For all plots, statistical enrichment (two-sided Paired Student’s *t* test) is shown above. Non-significant (ns) *p* values are 0.6, 0.27, and 0.26 for replicates 1, 2, and 3, respectively. Means, quartiles, and outlier limits (1.5 × interquartile range) are indicated by the median line, box and whiskers, respectively.

To directly determine if chromatin interactions, which are often controlled by the architectural protein CTCF, mediate γH2Ax propagation, we used Cas9-RNPs to remove, from the *Rag2*^−/−^ v-abl line, a CTCF motif positioned only 3 kb from the *Myc* promoter (5′CTCF KO, Fig. 4a and Supplementary Fig. 5A). We then verified depletion of CTCF binding at the targeted motif by CR-seq (Fig. 4a). Comparison of Hi-C data from WT and 5′CTCF KO cells revealed reduced contacts throughout the *Myc*-TAD in the mutant line (Fig. 4b–d, *p* < 0.05 HiCcompare, *M* test), a conclusion consistent with previous studies using cells lacking this CTCF motif^31^. Following RNP-induced damage at the *Myc* promoter, 5′CTCF KO cells had reduced γH2Ax at distal sites within the *Myc*-TAD, when compared with WT cells. Indeed, subtraction of γH2Ax or V.4C data revealed comparable shifts between the interactome and γH2Ax profiles (Fig. 4c). To further compare interactome and chromatin datasets, we quantified sequencing data in 10 kb bins, representing the Hi-C resolution, across the *Myc-*TAD or neighboring 5′ TAD. We observed consistently less γH2Ax across the KO TAD, correlating well with lower interaction intensities (Fig. 4d). Similar results were observed when we directed a DSB to the super-enhancer (SE) at the 3′ end of the locus (Supplementary Fig. 5B, C). In contrast, neither interactions nor γH2Ax intensities in the KO cells differed significantly for the 5′ TAD, suggesting that removal of this single CTCF site did not remove the topological border. Thus, deletion of the *Myc* 5′CTCF site perturbs interactions of the promoter and SE within its encompassing TAD, leading to a commensurate reduction of γH2Ax deposition following a DSB.

### DSBs at TAD borders extend γH2Ax domains bidirectionally

**Fig. 5:**
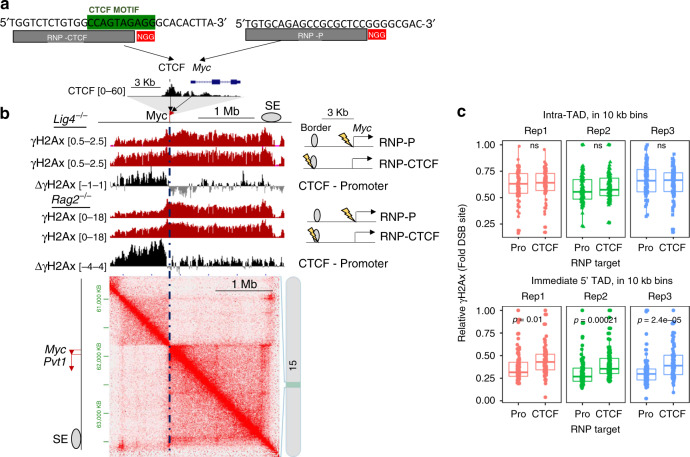
DSBs at a CTCF motifs propagate γH2Ax bidirectionally. **a** Guide target sequences and relative locations. Top: Genomic sequence of the upstream *Myc*-CTCF (RNP-CTCF) site or promoter (RNP-P) with target sites (gray with red NGG) and CTCF motif (green) labeled. Bottom: Zoom-in of the *Myc* promoter and 5′CTCF region, which encompasses the TAD border. Black track shows CTCF binding (RPKM, *n* = 1). **b** γH2Ax following DSBs targeted to the TAD border. Top: UCSC snapshot showing values for γH2Ax CR-seq (red, RPKM, representative of three independent replicates) and V.4C (gray, interaction score, *n* = 2 merged independent replicates). Cartoons to the right of tracks show relative regulatory element and DSB sites (lightning bolts). γH2Ax subtraction, labeled as Δ tracks (RNP-CTCF—RNP-P), show enrichment for the CTCF RNP in black and the promoter RNP in gray. Bottom: JuiceBox Browser representation of Hi-C data from *Lig4*^−/−^ cells for the *Myc* region (*n* = 2, merged independent replicates). **c** γH2Ax 2 h after 63–12 cells were nucleofected with RNP-*Myc*-P or RNP-CTCF, quantified in 10 kb bins across either the *Myc*-containing TAD or immediate 5′ TAD. Each γH2Ax dataset was first normalized to RPKM values at the DSB-containing bin. Colors represent independent replicates. Statistical enrichment (two-sided Paired Student’s *t* test) is shown above (ns non-significant). Means, quartiles, and outlier limits (1.5 × interquartile range) are indicated by the median line, box and whiskers, respectively.

Our findings with mutants lacking an architectural element in the *Myc* locus spurred us to test how γH2Ax distribution is impacted when a DSB occurs precisely at a CTCF motif within a topological border. In contrast to CTCF mutant cells, in which a new interactome is formed following deletion of the architectural element (see Fig. 4b), targeting a DSB to the CTCF site itself would examine γH2Ax propagation in cells with wild-type chromosomal contacts. For this purpose, we targeted an RNP to the same CTCF site, which is associated with the *Myc* 5′ TAD border (*Myc*-CTCF), using nucleofection of v-abl cells. We then compared γH2Ax between the *Myc*-CTCF and *Myc*-P breaks, the latter of which was situated only 3 kb downstream (Fig. 5a). Strikingly, a DSB occurring within the *Myc*-CTCF site propagated γH2Ax into both the *Myc-* and neighboring 5′-TAD (Fig. 5a, b), whereas γH2Ax was largely confined to the *Myc*-TAD following a break in the promoter. γH2Ax profiles within the *Myc*-TAD were not significantly different when comparing *Myc*-P and *Myc*-CTCF breaks (Fig. 5b, c). Similarly, DSBs at sites located within 40 bp of the CTCF motif (5′ or 3′), potentiated γH2Ax spreading into adjacent TADs, while having little impact on DDR platforms over the *Myc*-TAD (Supplementary Fig. 6A, B).

We observed similar multi-TAD γH2Ax propagation when additional CTCF sites corresponding to TAD borders were targeted by RNP nucleofection, either at the *Tcrb*-TAD (RNP-Vβ1 CTCF site) or the *Rasl10b*-TAD (RNP-*Rasl10b*) (Supplementary Fig. 6C). We conclude that CTCF-containing TAD borders can contribute as γH2Ax insulators for DSBs occurring at sites within a TAD, but lesions at the borders themselves allow for spreading of the DDR platform into two adjacent TADs. This is in sharp contrast to DSBs introduced even a short distance from a TAD border (e.g., at *Myc*-P or -SE), which generates relatively asymmetric γH2Ax domains, with a short and long DDR platform on each side of the broken chromosome (Model, Supplementary Fig. 6D).

## Discussion

It has been appreciated for some time that DSBs generate γH2Ax and a DDR platform encompassing a large swath of the neighboring genome, in which repair factors are concentrated and transcription is repressed^21,36^. We now find that γH2Ax landscapes reflect the basal interactome of a DSB site, whose boundaries often, but do not always, correspond to those of its native TAD. In this regard, TAD boundaries are defined statistically using relative insulation scores; thus, a called border does not absolutely exclude interactions between neighboring compartments, as observed, for example, at *Tcrb*. Indeed, recent high-resolution microscopy has visualized γH2Ax and 53BP1 foci as single and multi-TAD rings surrounding their DSB^22^. Mechanistically, chromatin contacts might compartmentalize the spread of γH2Ax, spatially concentrating DDR repair factors, perhaps via 53BP1-dependent phase separation^37^. The spatially-defined DDR compartments may simultaneously restrict separation of DSB ends to facilitate their efficient re-ligation. The contact-dependent nature of γH2Ax domains suggests that recruitment of ATM and DNA-PKc may be restricted to a DSB-proximal region^38^, which then phosphorylates H2Ax via physical contacts, rather than by linear propagation along the chromosome. The contact-dependent model (Supplementary Fig. 6D) would also explain contours we observed in DDR platforms; certain regions within a domain would remain free of γH2Ax due to limited interactions with the site that suffered damage. Therefore, although TADs define the same γH2Ax borders in a wide array of cell types, the profiles of this modification within DDR platforms will differ, depending on the actual DSB site and the cell type-specific patterns of chromosomal contacts.

Another important finding of our study is that DSBs can generate γH2Ax domains of widely divergent symmetries. We find that γH2Ax spreads symmetrically from breaks incurred near the center of a TAD, whereas a DSB near a TAD border leads to one short and one long DDR platform. This is in sharp contrast to our finding that DSBs at TAD borders generate long γH2Ax domains that spread throughout the two adjacent TADs, which often alternate between euchromatic and heterochromatic states. Prevailing evidence indicates that DSBs occurring in euchromatic regions favor end resection and HR, while heterochromatin favors NHEJ pathways^39,40^. It remains unclear how the hybrid γH2Ax domains formed by DSBs at TAD borders would direct repair; however, we would point out that many cancer types are characterized by TAD border mutations^41^.

Aside from damage at CTCF-containing TAD borders, wherein γH2Ax propagation becomes bidirectional, we find that the distribution of this DSB-induced modification largely reflects the pre-DSB interactome. Indeed, interactions observed in intact chromosomes are modestly enhanced throughout γH2Ax domains following a persistent DSB (see Supplementary Fig. 3). This observation suggests that mechanisms involved in establishing chromosomal contacts are preserved, even in persistently damaged loci. One such mechanism is loop extrusion, during which cohesin drives the formation of progressively larger chromatin loops until it stalls at TAD boundaries or convergent CTCF sites^42^. Indeed, loop extrusion is likely active on severed alleles during immunoglobulin class switch recombination, wherein it is required to align switch regions^43^. Conversely, defects in extrusion, or the TAD architecture, may compromise normal DDR signaling, a hypothesis that remains to be tested. A remaining unknown, however, is how the loop extrusion mechanism following a DSB could generate domains with large γH2Ax voids, which were observed at multiple loci in our study.

Given that γH2Ax is the platform for break stabilization, likely via mechanisms involving 53BP1 retainment^11^, we predict that the location of damage relative to a TAD border may also contribute to the stability of a broken chromosome. DSBs close to, but not at a TAD border (i.e., the *Myc* promoter), may have reduced stability due to highly asymmetric γH2Ax domains. Indeed, DSBs in this border-proximal *Myc* region are involved in chromosomal translocations associated with lymphocytic malignancies. Moreover, structural variants, especially those resulting from chromothripsis, are enriched at TAD borders in many types of cancer^44,45^. TAD-proximal DSBs may leave one chromosome end relatively unprotected by 53BP1, especially when the break persists, which could lead to extensive end resection and/or drifting of the two chromosome fragments^46,47^. Importantly, sites lying close to topological borders are common sources of damage, especially when the relief of ongoing torsional stress in chromosomes is inhibited. Such a scenario occurs when topoisomerase poisons are employed as chemotherapeutics, and can lead to therapy-associated leukemia^48,49^. Thus, contact-driven mechanisms for generating DDR platforms are likely to be critical determinants of genome integrity in the wake of natural or agent-induced DSBs.

## Methods

### Mouse models

Bcl2^tg^;IgH^tg^ *Rag*^−/−^, and *Artemis*^−/−^ mouse strains have been described previously^17^. DN thymocytes and CD19^+^ pre-B cells were isolated from 6-week-old animals, enriched for lymphocytes using an Ammonium–Chloride–Potassium lysing buffer (Thermo Fisher), and selected with microbeads (Miltenyi Biotec), based upon established protocols^50^. All animal studies were reviewed and approved by the Washington University Animal Review Board.

### Cell lines

V-abl pro B cell lines 63–12, *Lig4*^−/−^ and *Lig4*^−/−^FOK1-ZFN were created previously^25^. For creation of 5′CTCF KO cells, 63–12 cells were nucleofected with RNPs surrounding the *Myc*-CTCF site by 75 bp (see Supplementary Table 1 for sequences and locations), and subclones were screened for deletion using PCR (see Supplementary Table 1 for oligo sequences). *Rag1*^−/−^ thymoma line p5424 was created previously^51^. All cells were maintained in RPMI with 10% fetal bovine serum, antibotics and beta-Mercaptoethanol.

### RNP formation and nucleofection

Cas9-RNP complexes were formed by complexing 100 μM Cas9 (Berkley MacroLabs) with 200 μM in vitro transcribed guide RNA (GeneArt Precision gRNA synthesis kit, Invitrogen) for 20 min on ice in 10 μl RNase-free 1× Cas9 complexing buffer (20 mM HEPES, 100 mM NaCl, 5 mM MgCl2, 0.1 mM EDTA. pH 6.5). While complexing, cells were washed 2× in ice-cold PBS at 100 × *g*. Cells were resuspended in ice-cold 100 μl final volume Chikabuffer 1^52^ and nucleofected in a 1D-nucleofector (program X-001). Immediately following nucleofection, cells were transferred to pre-warmed/equilibrated 10% serum RPMI for indicated time points. Between uses, cuvettes were washed in water, 0.1 N HCL and 70% EtoH.

### Chip-Seq

ChIP-seq and native ChIP-seq was performed as follows: For ChIP-seq, 3 × 10^6^ cells were fixed for 5 min at room temperature, quenched with glycine, washed, and sonicated in a Diagenode Bioruptor Plus to approximately 140 bp (60 cycles). Cleared extracts were incubated with protein A- Dynabeads pre-complexed with antibody (Invitrogen, 10001D) overnight at 4 °C. Beads were then washed twice with each of the following buffers: low, high, LiCl and TE wash buffers. Samples were eluted using a sodium bicarbonate buffer at 55 °C and purified using a PCR cleanup column (see Supplementary Table 1). For native ChIP-seq, nuclei were isolated using a Nuclei Isolation Kit (Sigma, NUC101), then digested with MNase (1:10 dilution of NEB biolabs M0247S) for 5 min at room temperature in MNase digestion buffer. Cleared lysates were further processed identically to the cross-linked protocol above.

### Cut and Run-Seq

For Cut and Run-seq 500 k cells were processed as described^28,53^. Cells were washed 1× with cut and run wash buffer (20 mM HEPES, pH 7.5, 150 mM NaCl, 0.5 mM spermidine), bound to activated ConA beads (Bangs Laboratories BP531), permeabilized in digi buffer (wash buffer + 0.002% digitonin), incubated with antibodies (1 μg/CR in digi buffer), washed in digi buffer, incubated with pA-MN (gift from Heinkoff Laboratory, currently EpiCypher 15-1016), and washed in digi buffer. Following the final wash, cells were washed with ice-cold low salt wash buffer (20 mM HEPES, pH 7.5, 0.5 mM spermidine, 0.002% digitonin) and digested using MNase digestion buffer (3.5 mM HEPES pH 7.5, 10 mM CaCl2, 0.002% digitonin) for 25 min on ice. Solubilized chromatin was released using an isosmotic stop buffer (170 mM NaCl, 20 mM EGTA, 0.05% Digitonin, 20 µg/ml glycogen, 25 µg/ml RNase A, 2 pg/ml S. cerevisiae fragmented nucleosomal DNA) and was collected using a PCR cleanup kit column (EZbioresearch M1001). All antibody incubations and digitonin washing steps were performed for 5 min at room temperature and included protease, phosphatase and deacetylase inhibitors (Roche).

### Library preparation

For library preparation ChIP-, 4C or CR-DNA products were incubated with an end repair master mix (1× T4 ligation buffer, dNTP, ATP, T4 PNK, T4 DNA Pol and TAQ DNA polymerase) and incubated as follows: 12 °C for 15 min (end polishing), 37 °C for 15 min (5′ end phosphorylation), and 58 °C for 1.5 h (dA tailing). Polished libraries were purified using Ampure XP beads and ligated to either NEBNext Dual Index (NEB) or Illuminia TruSeq adapters. Libraries were size selected and enriched by PCR.

### Hi-C SEQ

Hi-C was performed as follows, based upon as in-situ protocols^54^. Briefly, 5 × 10^6^ formaldehyde-cross-linked cells were lysed on ice for 15 min with 250 μl of ice-cold Hi-C lysis buffer (10 mM Tris-HCl pH8.0, 10 mM NaCl, 0.2% Igepal CA630) containing protease inhibitors (Roche). Chromatin was digested using DpnII (100 U) at 37 °C for 6 h. The digested DNA ends were then filled and marked with biotin using Klenow, followed by ligation with T4 DNA ligase. After reversing the cross-links, DNA was fragmented using a Covaris E220 Evolution Sonicator followed by size-selection for 300–500 bp using AMPure XP Beads (Beckman Coulter). DNA end repair was performed using NEBNext Ultra II DNA Library Prep Kit according to the manufacturer’s instructions using 1 μg of the Hi-C DNA. Adapter-ligated DNA was then selected for 300–400 bp using AMPure XP beads and the biotinylated DNA fragments were pulled down using MyOne Streptavidin T1 beads (Life Technologies). The final Hi-C library was generated with 5 PCR cycles using the NEBNext Ultra II DNA Library Prep Kit and NEBNext Dual Index primers (NEB) for Illumina sequencing.

### Sequencing and analysis

Libraries were constructed as described^55^. Finished libraries were sequenced using Illumina HiSeq2000 instrument (ChIP-, CR- and 4C-seq: 50-bp single-end) or NovaSeq S1 instrument (Hi-C: 101-bp paired-end: 500M-1B reads). Unique reads were aligned to the reference build (GTCm387/mm9) using TopHat and Bowtie2. RPKM values were obtained using Deeptools^56^. HI-C Reads were processed using Juicer pipeline^57^ and visualized using JuiceBox or HiCExplorer^35^. Replicate HI-C data were tested for concordance using HiCExplorer’s hicCorrelate (*r*^2^ > 0.75, Pearson’s correlation) and merged. The UCSC Genome Browser tack collection hub was used to generate subtraction plots, correlation statics, and to visualize tracks. For all track visualization, data binning is presented as mean signal.

### Statistical analysis

For HiCcompare^58^ *Z*-statistic tests, JuiceBox.HiC files were extracted, and 25 kb binned using straw, then uploaded to R and processed for significance with the HiCcompare pipeline base settings. Pearson’s correlations were generated using the UCSC genome browser Table Browser Correlate function, using settings for 25 kb bins and a 5 Mb interval surrounding the DSB site. Fisher’s Exact Tests were performed on TAD and γH2Ax peak files using bedtools fisher. TADs and TAD insulation scores were called using HiCExplorer’s hicFindTADs^35^ function with 20–200 kb bins, with the following settings: —thresholdComparisons 0.01—delta 0.2. Peak files were generated using HOMER’s CallPeaks-histone, with a non-targeting guide as the input control.

### Reporting summary

Further information on research design is available in the Nature Research Reporting Summary linked to this article.

## Supplementary information

Supplementary Information

Reporting Summary

## Data Availability

Next-generation sequencing data are available at the Gene Expression Omnibus under accession number GSE150384. All other data are available from the authors upon reasonable request.
