# Supplementary material for: DNA double-strand breaks induce H2Ax phosphorylation domains in a contact-dependent manner
Source: Nat Commun. 2020 Jun 22;11:3158. doi: 10.1038/s41467-020-16926-x (PMC7308414; doi:10.1038/s41467-020-16926-x)
Supplement: Supplementary file 1 — Supplementary Information [file 41467_2020_16926_MOESM1_ESM.pdf]

Supplementary Information

"DNA Double-Strand Breaks Induce H2Ax Phosphorylation Domains in a Contact-Dependent Manner"

Collins et al.

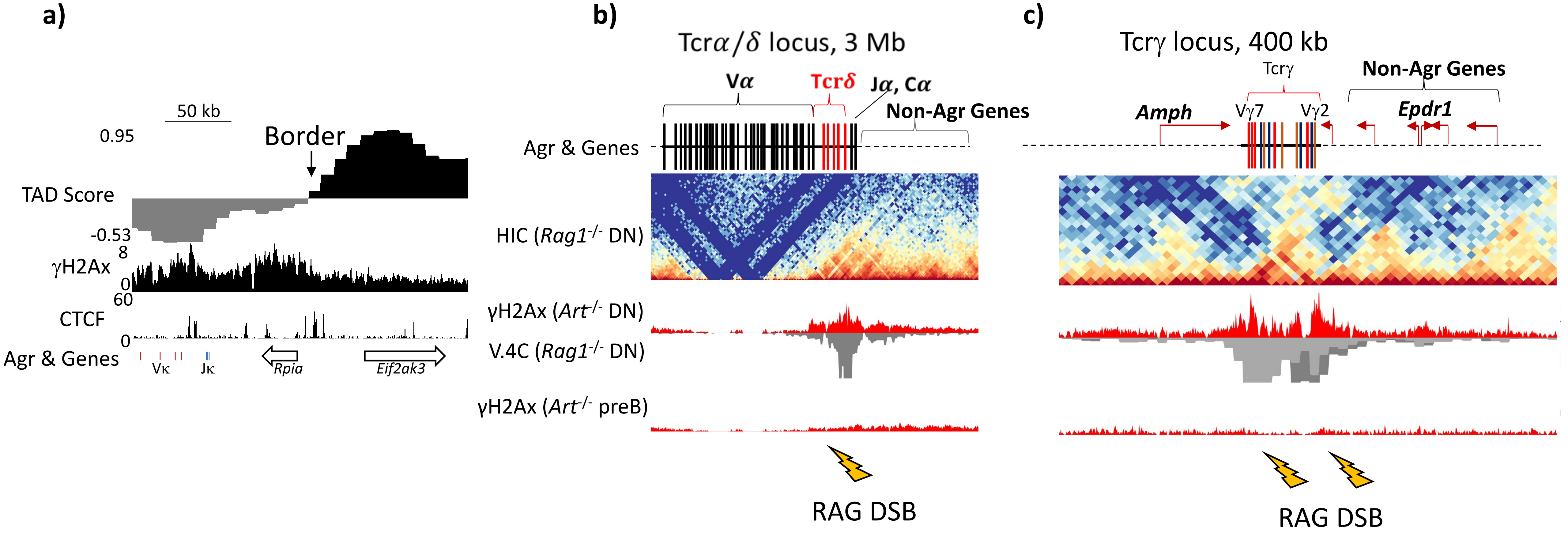

Supplementary Figure 1: Supplemental data related to Figure 1.

A) UCSC genome browser snapshot showing the 3' *Igk* TAD border. Data are derived from *Lig4*$^{-/-}$ G1-arrested v-abl pre-B cells following Imatinib treatment (72h). Tracks show RPKM-normalized histograms for γH2Ax ChIP-seq (RPKM mean of three independent replicates), CTCF Cut and Run-seq (n=1), and relative TAD insulation scores, derived from 40 kb bins (blue lines) or 20-100 kb bins (grey lines). The *Igk* TAD border position is indicated above. **(B-C)** HiCExplorer PlotTADs representation of **(B)** *Tcrad* and **(C)** *Tcrg* Hi-C contacts (n=2, merged independent replicates), γH2Ax CR-seq (Red, RPKM, representative of 2 independent replicates), and V.4C data (grey, interaction counts, n=2, merged independent replicates), from the indicated cell types. Each panel includes diagrams indicating antigen receptor loci, and genes on top, and the DSB location (lightning bolt) at the bottom. For *Tcrg*, the two most commonly rearranged V segments were used as V.4C viewpoints (grey and dark grey).

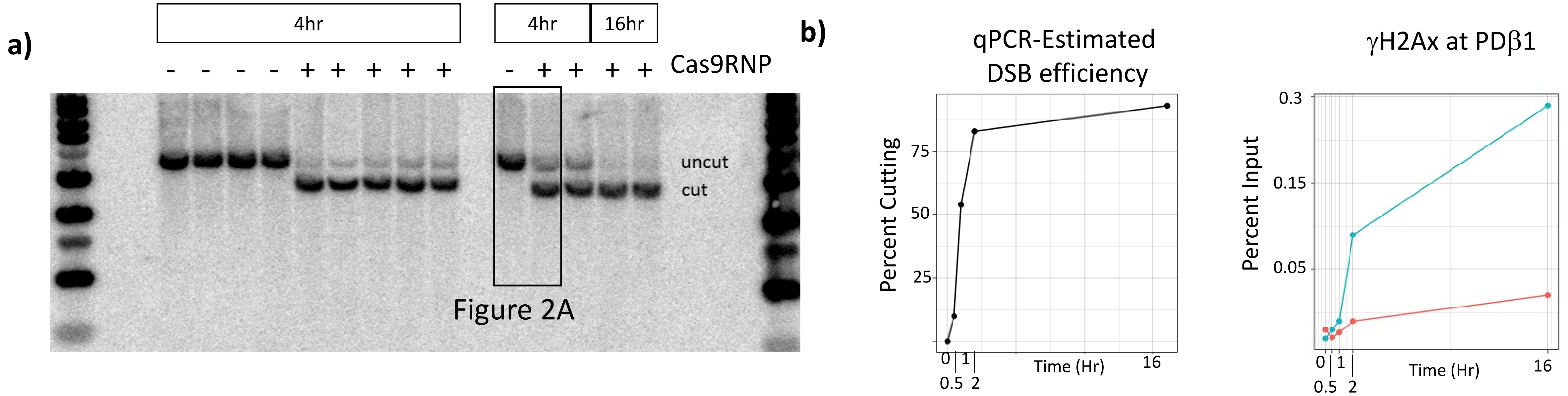

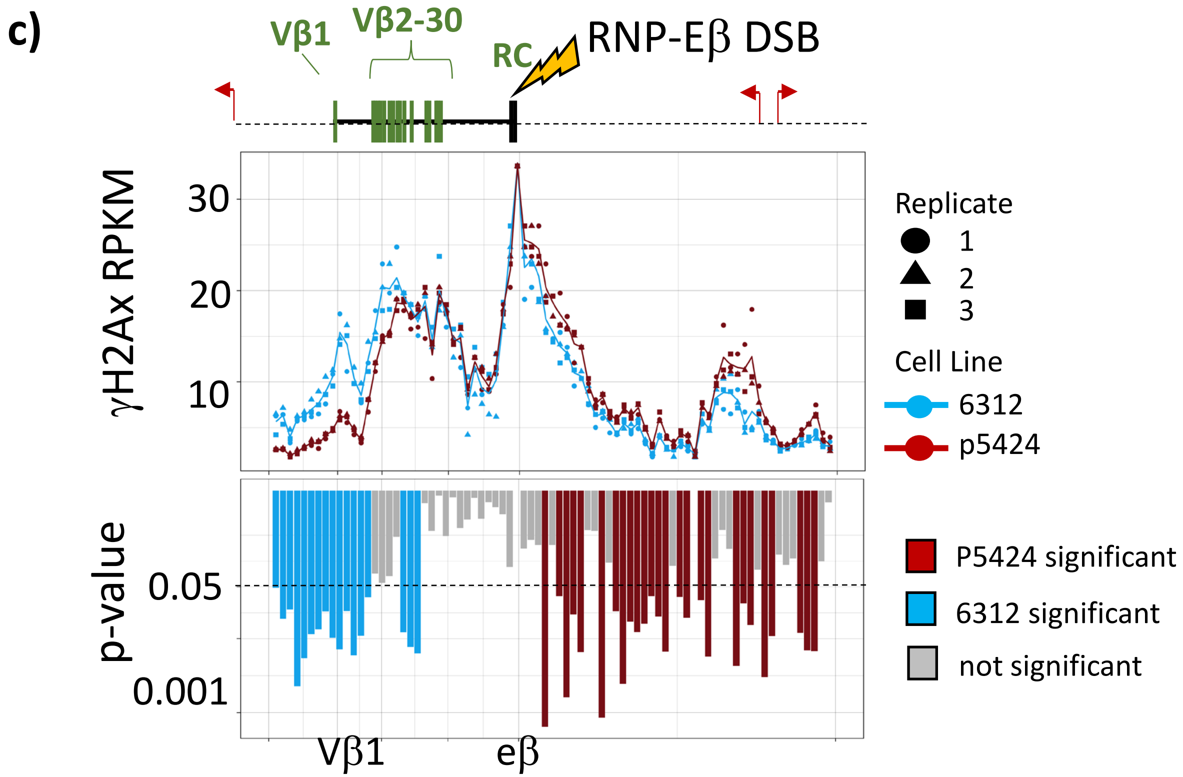

Supplementary Figure 2: Supplemental data related to Figure 2.
A) Southern blotting analysis, as described in Figure 2. Data are shown for independent replicates, as well as for an extended, 16-hour time point. Black box indicates a cropped section used for figure 2A. **B)** Left: Proportion of cut alleles as a function of time post-nucleofection was calculated by relative qPCR. For this assay, any negative value is shown as 0% cutting. Right: G1-arrested *Lig4*$^{-/-}$ v-abl cells were nucleofected with RNP-eβ (blue) or an RNP for a control genomic region on a separate chromosome (red) and processed for γH2Ax ChIP-qPCR using oligos targeting PDβ1. Relative γH2Ax abundance was calculated using percent input. **C)** Dot and line plot showing γH2Ax in 25 kb bins across *Tcrb*, two hours after pro- (63-12: blue) or pre- (p5424: red) lymphocyte cell lines were nucleofected with an RNP-Eβ. Dots show three independent replicates while lines indicate mean γH2Ax values. The locations of gene segments, regulatory elements and RNP target (lightning bolt) are shown at the top. Statistical significance and P values (Student's Paired T test) for each 25 kb bin is shown on bottom (blue significantly enriched in 6312, red significantly enriched in p5424).

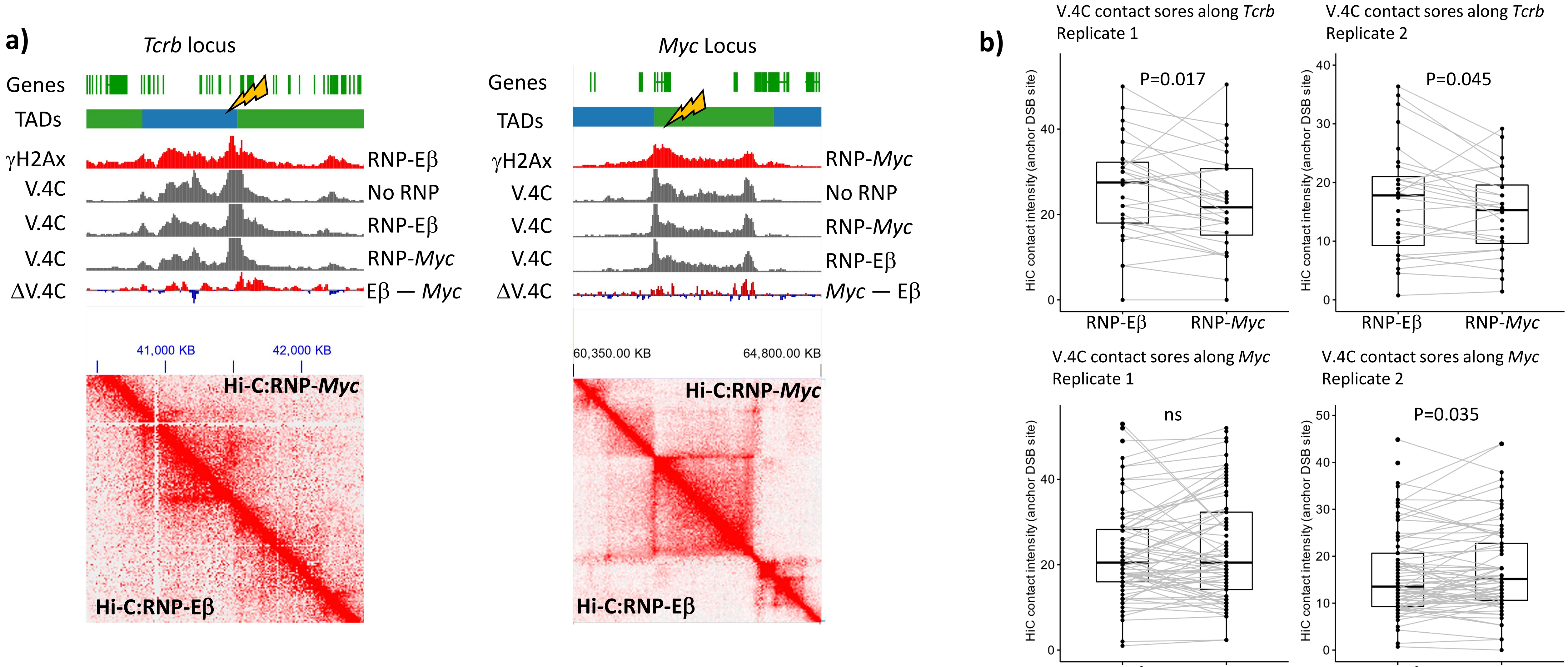

Supplementary Figure 3: Supplemental data related to Figure 2.

A) Juicebox browser snapshots showing the (left) *Tcrb* or (right) *Myc* loci in cells nucleofected with the indicated RNP complexes, or control cells that were not nucleofected. Locations of genes, gene segments, and TAD locations are shown at the top of each panel. RNP target locations and V.4C data extraction viewpoints are shown as lightning bolts. Tracks represent values for γH2Ax CR-seq (Red, RPKM, representative of three independent experiments) or V.4C (Grey, representative of two independent experiments). Subtraction plots show differences in mean V.4C signal from cells receiving RNP-Eβ versus RNP-*Myc*. Bottom: JuiceBox Hi-C plots after coverage-normalization, derived from G1-arrested *Lig4*$^{-/-}$ v-abl cells receiving RNPs targeted to *Myc* (top right) or Eβ (bottom left). **B)** *Myc* promoter or Eβ interactome derived from Hi-C data of cells two hours after RNP-Eβ or RNP-*Myc* nucleofection, quantified in 25 kb bins across the targeted TAD. Relative interactome measurements have been normalized using coverage normalization and down sampling. For all plots, statistical enrichment (two-sided Paired Student's T test) is shown above. Means, quartiles, and outlier limits (1.5 x interquartile range) are indicated by the median line, box and whiskers, respectively. Grey lines indicate paired bins. Genomic intervals with over 50 interaction counts, which correspond to nearest neighbors, are not shown.

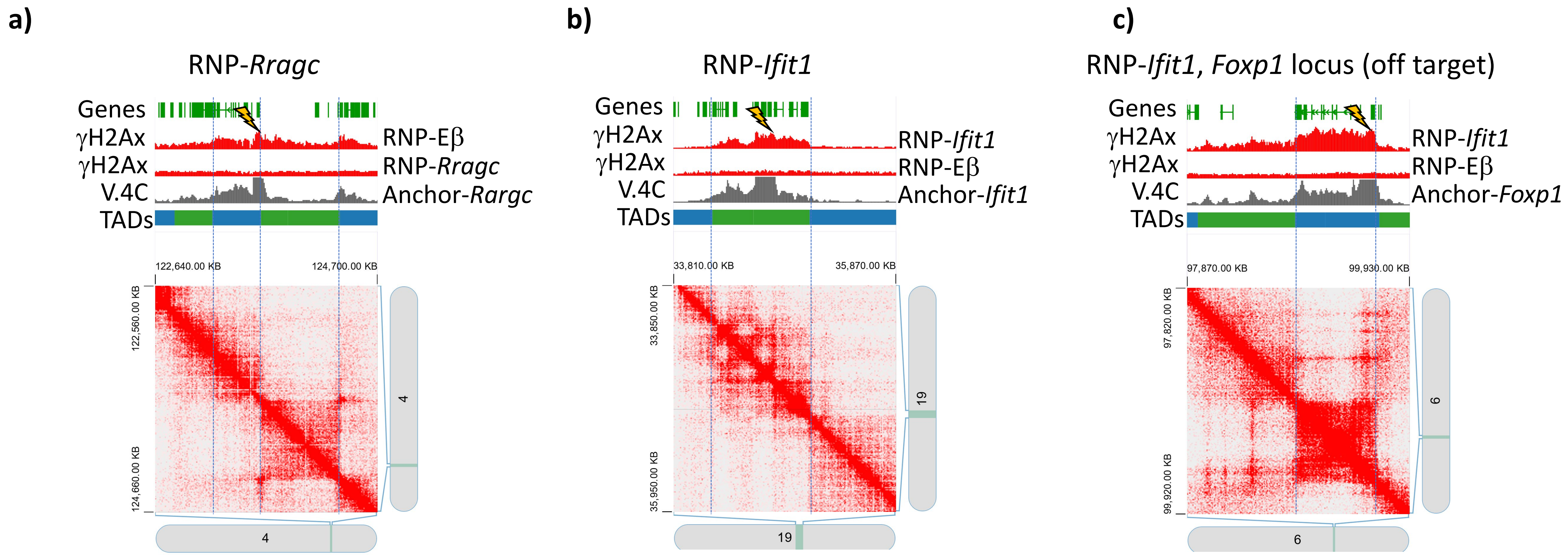

Supplementary Figure 4: Supplemental data related to Figure 3.
Juicebox browser snapshots showing the *Rragc* **(A)**, *Ifit1* **(B)**, or *Foxp1* **(C)** loci nucleofected with the indicated RNP complexes, or a control RNP targeted to Eβ (Representative of 2 independent replicates, each). Data are represented as in Figure 3.

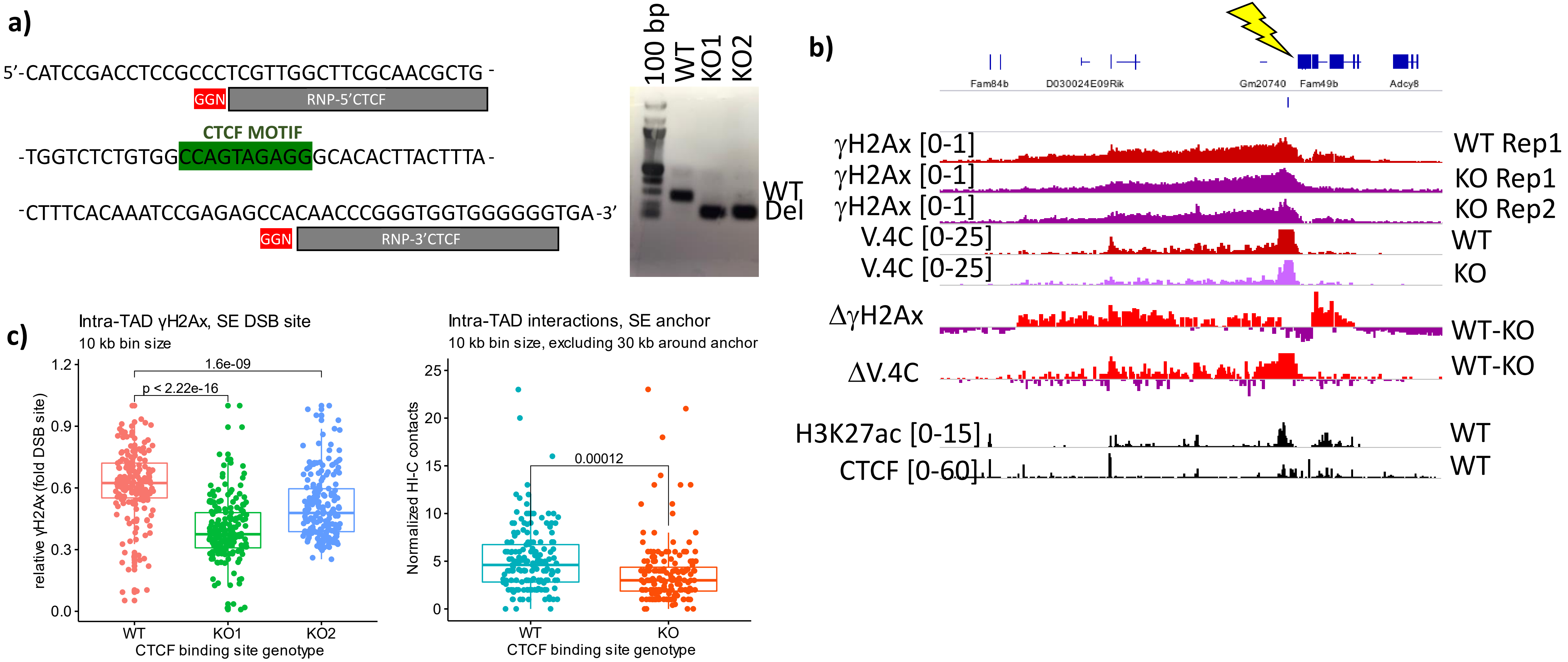

Supplementary Figure 5: Supplemental data related to Figure 4

A) Left: Genomic sequence of the upstream *Myc* CTCF site with RNP target sites (grey with red NGG) and CTCF motif (green) labeled. RNPs labeled 5' and 3' CTCF were used for subclone creation. Right: PCR showing deletion of the upstream *Myc* CTCF site Gel image is representative of at least two independent genotyping PCRs. **B)** IGV snapshot, as described in Figure 4, showing the *Myc* locus in 63-12 cells with and without the 5' CTCF binding motif. Tracks representing γH2Ax data were normalized to the DSB-containing bin. For subtraction plots of γH2Ax or V.4C data, red represents WT enrichment, while purple represents KO enrichment. Independent replicates are indicated on the figure. RPKM-normalized CTCF and H3K27ac CR-seq tracks are shown on the bottom (n=1, each). **C)** *Myc* super-enhancer interactome and γH2Ax derived from 5'CTCF WT or KO cell lines, quantified in 10 kb bins across the *Myc*-containing TAD. Left: γH2Ax two hours after 5'CTCF WT or KO cells were nucleofected with RNP *Myc*-SE. Each γH2Ax dataset was first normalized to RPKM values at the DSB-containing bin. Replicates are indicated on the figure. Right: Comparison of *Myc* promoter interactomes, derived from Hi-C data, across the *Myc*-containing TAD of indicated genotypes, without RNP nucleofection. Relative interactome measurements have been normalized using coverage normalization. For all plots, statistical enrichment (two-sided Paired Student's T test) is shown above. Means, quartiles, and outlier limits (1.5 x interquartile range) are indicated by the median line, box and whiskers, respectively.

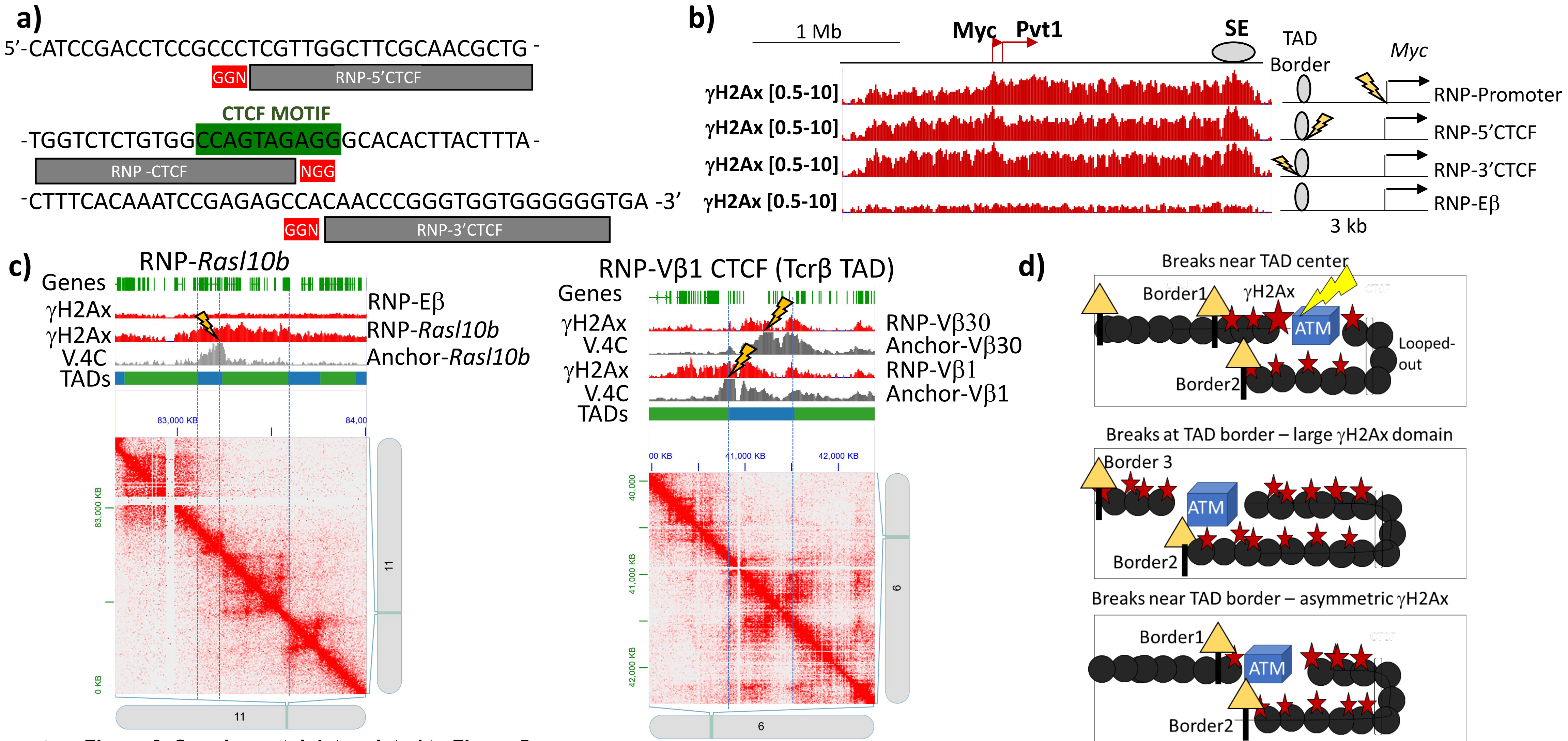

Supplementary Figure 6: Supplemental data related to Figure 5

A) Genomic sequence of the upstream *Myc* CTCF site with RNP target sites (grey with red NGG) and CTCF motif (green) labeled. RNPs labeled 5' and 3' CTCF were used for RNP targeting in Supplementary Figure 6, while RNP labeled CTCF was used for Figure 5. **B)** γH2Ax following DSBs targeted to the TAD border. UCSC snapshot show values for γH2Ax CR-seq (red, RPKM, representative of two independent experiments). Cartoons to the right of tracks show relative regulatory element and DSB sites (lightning bolts). **C)** Juicebox browser snapshots showing the *Ras10b* and *Tcrb* loci, nucleofected with the indicated RNP complexes or a control RNP targeted to eβ. Top: Scale bar, and gene locations (green). Middle: Tracks represent mean γH2Ax CR-seq (red, scaled as min-max, representative of 2 independent replicates) and V.4C (grey, scaled as 0-50 interaction score, n=2 merged independent replicates). RNP target sites and V.4C anchors are shown as dashed blue lines. Bottom: JuiceBox Hi-C plots showing coverage normalized interaction scores. Relevant TADs are highlighted with dashed boxes. **D)** Model of γH2Ax domain formation via chromosomal contacts. Top: Intra-TAD DSBs lead to propagation of γH2Ax via contacts with the break site; Middle: DSB at a TAD border leads to spreading of γH2Ax throughout both adjacent TADs; and, Bottom: DSBs near a TAD border generate highly asymmetric γH2Ax domains on each side of the break, where spreading on one side is blocked by the proximal CTCF site.

Supplementary Table 1

Key resources table listing the reagents and datasets used in this study

REAGENT or RESOURCE	SOURCE	IDENTIFIER
Antibodies		
Anti-H2Ax (3 μg/ChIP)	Abcam	Ab11175
Anti-phosphoH2Ax (3 μg/ChIP or 1 μg/Cut and Run)	Millipore	05-636
Anti-CTCF (1 μg/Cut and Run)	Rockland	600-401-C42
Anti-H3K27Ac (1 μg/Cut and Run)	Abcam	Ab4722
Rabbit anti Mouse (1 μg/Cut and Run)	Invitrogen	31188
Chemicals, Peptides, and Recombinant Proteins		
Digitonin	EMD Millipore	300410
Spermidine	Sigma-Aldrich	S2501
Protein A Dynabeads	Invitrogen	10001D
ConA Microbeads	Bangs Laboratory	BP531
Protein A Mnase	Skene et al. 2018	
MNase	NEB	M0247S
Cas9-NLS	Berkley Macrolabs	
ACK lysing buffer	ThermoFisher	A1049201
TRizol	Invitrogen	15596026
T4 DNA Polymerase	New England Biolabs	M0203L
Klenow DNA Polymerase	New England Biolabs	M0210L
T4 PNK	New England Biolabs	M0201L
Klenow (3'-5' Exo-)	New England Biolabs	M0212L
Quick DNA Ligase	New England Biolabs	M2200L
Phusion HF Master Mix	New England Biolabs	M0531L
Complete, EDTA-Free PI Cocktail	Roche	11873580001
Critical Commercial Assays		
GeneArt™ Precision gRNA Synthesis Kit	ThermoFisher	A29377
Agencourt AMPure XP	Beckman Coulter	A63880
RNeasy Plus Micro kit	Qiagen	74034
Nextera DNA Library Preparation Kit	Illumina	FC-121-1030
Nextera Index Kit	Illumina	FC-121-1011
NEBNext ChIP-Seq Library Prep Master Mix Set for Illumina	NEB	E6240L
EZ nuclei isolation kit	Sigma	NUC101-1KT
Low Bind DNA Tubes	Ambion	AM12450
Deposited Data		
Raw and Analyzed Data	GSE150384	
Experimental Models: Cell Lines		
Lig4-/- FokI-ZFN Abl Cell	Lee et al. 2013	Lig4-/- FokI-ZFN
Lig4-/- Abl Cells	Helmink et al 2011	$Lig4^{-/-}$
$Rag2^{-/-}$ Rag1-/- Abl pro lymphocyte line	Ji Y et al. 2010	63-12
P5424 cells pre lymphocyte line	Mombaerts et al. 1995	P5424
Experimental Models: Organisms/Strains		

Artemis -/- Bcl2Tg mice	Bednarski et al., 2012	
Rag-/- Bcl2-Tg mice	Bednarski et al., 2012	
Rag-/- Mice	JAX Lab	002216 - B6.129S7-Rag1<tm1Mom>/J
**Oligonucleotides**		
RNP-Fam49b genomic target	GATGTACAGGGTAACGGCGA	mm9 chr15:63829296-63829978
RNP-MYC Pro genomic target	GTGTGCAGAGCCGCGCTCCG	mm9 chr15:61816821-61817283
RNP-MYC SE genomic target	GGATGCCTCTCACTGAGTCA	mm9 chr15:63,510,641-63,510,773
RNP-MYC CTCF motif genomic target	GTGGTCTCTGTGGCCAGTAG	mm9 chr15:61814727-61815220
RNP-FAM84b genomic target	GCTCGCCACGCACCTGCACC	mm9 chr15:60,654,471-60,654,943
RNP-eBeta 5' genomic target	ACTGTCACGGTGATAGCTAG	mm9 chr6:41504605-41504606
RNP-eBeta 3' genomic target	AGTGGGAAGAGCGGTGATGT	mm9 chr6:41504799-41504800
RNP-MYC CTCF 5' genomic target	AGCGTTGCGAAGCCAACGA	mm9 chr15:61815048-61815150
RNP-Vb30 genomic target	GTAGAATGTTAGGCAGGCAT	mm9 chr6:41166002-41166061
RNP-Agk genomic target	AGAAAGTAATTAAATGGCTA	mm9 chr6:40275167-40275304
RNP-eBeta genomic target	GATGTAAGATAGTTTAGATG	mm9 chr6:41504684-41504685
**Software and Algorithms**		
Bowtie2	Langmead and Salzberg, 2012	http://bowtie-bio.sourceforge.net/bowtie2/index.shtml
Samtools	Li et al., 2009	http://samtools.sourceforge.net/
Novoalign	Hercus et al, 2012	www.novocraft.com
Picard	MIT open license	https://broadinstitute.github.io/picard/
MACs2	Zhang Y et al, 2008	https://github.com/taoliu/MACS
DeepTools		http://www.biconductor.org
BEDTools	Quinlan A.R. and Hall I.M, 2010	bedtools.readthedocs.io
Microsoft Office		
ggplot2	Wickham H., 2016	http://ggplot2.tidyverse.org/
UCSC Genome Browser	Karolchik et al, 2003	https://genome.ucsc.edu/
HicCompare	Stansfield et al, 2018	http://www.biconductor.org
