# Supplementary material for: DNA double-strand breaks induce H2Ax phosphorylation domains in a contact-dependent manner
Source: Nat Commun. 2020 Jun 22;11:3158. doi: 10.1038/s41467-020-16926-x (PMC7308414; doi:10.1038/s41467-020-16926-x)
Supplement: Supplementary file 2 — Reporting Summary [file 41467_2020_16926_MOESM2_ESM.pdf]

nature research

Corresponding author(s): *Double-blind peer review submissions: write DBPR and your manuscript number here instead of author names.*

Last updated by author(s): *YYYY-MM-DD*

Reporting Summary

Nature Research wishes to improve the reproducibility of the work that we publish. This form provides structure for consistency and transparency in reporting. For further information on Nature Research policies, see our Editorial Policies and the Editorial Policy Checklist.

Statistics

For all statistical analyses, confirm that the following items are present in the figure legend, table legend, main text, or Methods section.

n/a	Confirmed	
☐	☒	The exact sample size (*n*) for each experimental group/condition, given as a discrete number and unit of measurement
☒	☐	A statement on whether measurements were taken from distinct samples or whether the same sample was measured repeatedly
☐	☒	The statistical test(s) used AND whether they are one- or two-sided<br>*Only common tests should be described solely by name; describe more complex techniques in the Methods section.*
☒	☐	A description of all covariates tested
☒	☐	A description of any assumptions or corrections, such as tests of normality and adjustment for multiple comparisons
☒	☐	A full description of the statistical parameters including central tendency (e.g. means) or other basic estimates (e.g. regression coefficient) AND variation (e.g. standard deviation) or associated estimates of uncertainty (e.g. confidence intervals)
☐	☒	For null hypothesis testing, the test statistic (e.g. *F*, *t*, *r*) with confidence intervals, effect sizes, degrees of freedom and *P* value noted<br>*Give P values as exact values whenever suitable.*
☒	☐	For Bayesian analysis, information on the choice of priors and Markov chain Monte Carlo settings
☒	☐	For hierarchical and complex designs, identification of the appropriate level for tests and full reporting of outcomes
☒	☐	Estimates of effect sizes (e.g. Cohen's *d*, Pearson's *r*), indicating how they were calculated

Our web collection on statistics for biologists contains articles on many of the points above.

Software and code

Policy information about availability of computer code

Data collection	Data from NGS sequencers were aligned using Novoalign (4.0), Bowtie2(2.4.1)
Data analysis	Microsoft Office (16.0) and open source software: Bowtie2 (2.4.1), Samtools (1.10), Hi-C Explorer (3.4.2), Picard (2.3.0), MACs2, Deeptools (3.3.0), BEDTools (2.29.2), ggplot2 (3.2.0), UCSC genome browser

For manuscripts utilizing custom algorithms or software that are central to the research but not yet described in published literature, software must be made available to editors and reviewers. We strongly encourage code deposition in a community repository (e.g. GitHub). See the Nature Research guidelines for submitting code & software for further information.

Data

Policy information about availability of data

All manuscripts must include a data availability statement. This statement should provide the following information, where applicable:

- Accession codes, unique identifiers, or web links for publicly available datasets
- A list of figures that have associated raw data
- A description of any restrictions on data availability

The datasets generated and analyzed during the current study are available in the GEO repository GSE150384

Field-specific reporting

Please select the one below that is the best fit for your research. If you are not sure, read the appropriate sections before making your selection.

☒ Life sciences ☐ Behavioural & social sciences ☐ Ecological, evolutionary & environmental sciences

For a reference copy of the document with all sections, see nature.com/documents/nr-reporting-summary-flat.pdf

Life sciences study design

All studies must disclose on these points even when the disclosure is negative.

Sample size	The minimal replicate number for genome-wide chromatin mapping was chosen as n=2. We determined this sufficient because of low variability observed between biologically independent replicates (greater than 90% peak overlap). γH2Ax mapping experiments were also tested between at least two different cell types, obtaining identical results. When this was not possible, n=3 was chosen because it was the minimal replicate number sufficient to ascertain statistics by T-test.
Data exclusions	Sequencing files were excluded for further analysis if they had no detectable signal above background, as determined by peak calling (MACS2).
Replication	We repeated key chromatin mapping experiments at least three times, using completely independent biological replicates. We also replicated γH2Ax mapping experiments (each n=2) across diverse cell types and experimental systems, obtaining highly consistent results. Failed sequencing reactions, determined as data without any significant signal over background noise via MACs2, were discarded and repeated. Data corresponding to one chromatin mapping experiment (Supplemental Figure 5C) was not returned in a timely manner due to a COVID19-related laboratory shut down. Hi-C experiments are cost-prohibitive and were carried out in duplicate. However, all attempts at Hi-C replication were successful, as determined using HiCExplorer's hicCorrelate between biologically independent samples (r2 > 0.75, pearson's correlation). All other experiments (Southern Blotting, qPCR and genotyping) were done at least three times and replicated successfully.
Randomization	As our study targeted specific mice or cell lines of known genotypes and tissue origin, randomization was not possible. Covariates, such as animal sex, age, and breeding facility; and cell line culture conditions were kept constant.
Blinding	Blinding was not possible in this study. It was obvious which datasets were derived from cells with double strand breaks (DSBs), and which were not. Further, the major readout of this study, genome wide γH2Ax mapping, only generated signal following DSB induction. HI-C data were independently analyzed by two bioinformaticians.

Reporting for specific materials, systems and methods

We require information from authors about some types of materials, experimental systems and methods used in many studies. Here, indicate whether each material, system or method listed is relevant to your study. If you are not sure if a list item applies to your research, read the appropriate section before selecting a response.

Materials & experimental systems

n/a	Involved in the study	
☐	☒	Antibodies
☐	☒	Eukaryotic cell lines
☒	☐	Palaeontology and archaeology
☒	☐	Animals and other organisms
☒	☐	Human research participants
☒	☐	Clinical data
☒	☐	Dual use research of concern

Methods

n/a	Involved in the study	
☐	☒	ChIP-seq
☒	☐	Flow cytometry
☒	☐	MRI-based neuroimaging

Antibodies

Antibodies used	Anti-H2Ax; Abcam Ab1175; lot GR269626-2; 3 µg/ ChIP-seq<br>Anti-γH2Ax; Millipore 05-636; lot:GR155796-2; 3 µg/ ChIP or1 µg/ Cut and Run<br>Anti-CTCF; Rockland 600-401-C42; lot: 26199; 1 µg/ Cut and Run<br>Anti-H3K27ac; Abcam Ab4722; Lot: 26199; 1 µg/ Cut and Run<br>Rabbit Anti-Mouse; Invitrogen 31188; Lot: QF2042603; 1 µg/ Cut and Run
Validation	All antibodies were purchased from commercial vendors, who provide validation information on their website. We performed independent validation as follows: Anti-H2Ax (Ab1175) and Anti-γH2Ax (Millipore 05-636) antibodies were validated by ChIP-seq against H2Ax knockouts. This analysis was not possible for antibodies targeting CTCF (Rockland 600-401-C42) and H3K27ac (Ab4722), as knockouts are lethal. Therefore, for validation we compared ChIP-seq tracks with established binding locations or modified regions, respectively. Rabbit anti-mouse (Invitrogen 31188) was validated for specificity by flow cytometry.

Eukaryotic cell lines

Policy information about cell lines

Cell line source(s)	A) P5424 cells (pre lymphocyte line): Used in lab for years. Originally Mombaerts et al. 1995 B) Rag-/- Abl (pro lymphocyte line): Used in lab for years. Originally Ji Y et al. 2010. C) Lig4-/- and Lig4-/- FokI-ZFN Abl Cell: Created on the floor (Lee et al. 2013)
Authentication	Cell line identity was verified as follows: 1) surface staining and flow cytometry (p5424 were verified as CD4+ & CD8+, while v-abl lines are CD19+); 2) all Lig4-/- v-abl lines were verified for ligase4 deficiency by PCR; 3) Cell lines lacking CTCF motifs were validated for a loss of CTCF motif sequence by genomic sequencing, and for a loss of CTCF binding determined by CTCF Cut and Run-seq; and 4) For FokI-ZFN cells, or Lig4-/- lines after RNP-nucleofection, inability to repair DSBs was verified by Southern Blotting.
Mycoplasma contamination	Cells were verified as Mycoplasma negative by a core facility at Washington University), and routinely tested
Commonly misidentified lines (See ICLAC register)	None

ChIP-seq

Data deposition

☒ Confirm that both raw and final processed data have been deposited in a public database such as GEO.

☒ Confirm that you have deposited or provided access to graph files (e.g. BED files) for the called peaks.

Data access links *May remain private before publication.*	The reviewers can download processed files with CyVerse: https://de.cyverse.org/de/?type=data&folder=/iplant/home/patlennycollins/gammaH2Ax
Files in database submission	ChIP, Cut and Run, and Hi-C data are deposited in GSE150384
Genome browser session (e.g. UCSC)	https://genome.ucsc.edu/cgibin/ hgTracks?db=mm9&lastVirtModeType=default&lastVirtModeExtraState=&virtModeType=default&virtMode=0&nonVirtPosition=&position=chr15%3A58483507%2D65583595&hgsid=754553795_ldSa4sMzpXKT4Lv3bNiGns9Eikgb

Methodology

Replicates	Two independent replicates were done for Hi-C experiments, checked for concordance, and merged for final visualization (to provide read depth). Key cut and run experiments were done in triplicate or duplicate, which are shown in the supplement.
Sequencing depth	ChIP-, 4C- and Cut and Run-seq experiments were sequenced to 25M reads on an Illuminia HiSeq. HiC experiments were sequenced to 500 M reads on an Illuminia Novaseq
Antibodies	Anti-H2Ax; Abcam Ab1175; lot GR269626-2; 3 µg/ ChIP-seq<br>Anti-γH2Ax; Millipore 05-636; lot:GR155796-2; 3 µg/ ChIP or1 µg/ Cut and Run<br>Anti-CTCF; Rockland 600-401-C42; lot: 26199; 1 µg/ Cut and Run<br>Anti-H3K27ac; Abcam Ab4722; Lot: 26199; 1 µg/ Cut and Run<br>Rabbit Anti-Mouse; Invitrogen 31188; Lot: QF2042603; 1 µg/ Cut and Run
Peak calling parameters	Peaks were called using using MACS2: macs2 callpeak -t [input file] -c [no DSB γH2Ax file side-by-side control] -F bam -g mm --outdir [output directory] -q 0.01 --broad
Data quality	Due to the unique nature of γH2Ax (signal on 0.5-3M only surrounding a DSB i.e < 0.001% of the genome) conventional quality metrics can not be used. Instead, we verified any peaks, compared to no DSB tracks, within 1 Mb of a DSB site.
Software	Microsoft Office (16.0), Novoalign (4.0) and open source software: Bowtie2(2.4.1), Samtools (1.10), Hi-C Explorer (3.4.2), Picard (2.3.0), MACs2, Deeptools (3.3.0), BEDTools (2.29.2), ggplot2 (3.2.0), UCSC genome browser
